# Oral Prion Disease Pathogenesis Is Impeded in the Specific Absence of CXCR5-Expressing Dendritic Cells

**DOI:** 10.1128/JVI.00124-17

**Published:** 2017-04-28

**Authors:** Barry M. Bradford, Boris Reizis, Neil A. Mabbott

**Affiliations:** a The Roslin Institute and Royal (Dick) School of Veterinary Sciences, University of Edinburgh, Easter Bush, United Kingdom; b Department of Pathology, New York University Langone Medical Center, New York, New York, USA; Rocky Mountain Laboratories

**Keywords:** prions, chemokines, intestine, dendritic cells, Peyer's patches, gastrointestinal infection, transmissible spongiform encephalopathy

## Abstract

After oral exposure, the early replication of certain prion strains upon stromal cell-derived follicular dendritic cells (FDC) in the Peyer's patches in the small intestine is essential for the efficient spread of disease to the brain. However, little is known of how prions are initially conveyed from the gut lumen to establish infection on FDC. Our previous data suggest that mononuclear phagocytes such as CD11c^+^ conventional dendritic cells play an important role in the initial propagation of prions from the gut lumen into Peyer's patches. However, whether these cells conveyed orally acquired prions toward FDC within Peyer's patches was not known. The chemokine CXCL13 is expressed by FDC and follicular stromal cells and modulates the homing of CXCR5-expressing cells toward the FDC-containing B cell follicles. Here, novel compound transgenic mice were created in which a CXCR5 deficiency was specifically restricted to CD11c^+^ cells. These mice were used to determine whether CXCR5-expressing conventional dendritic cells propagate prions toward FDC after oral exposure. Our data show that in the specific absence of CXCR5-expressing conventional dendritic cells the early accumulation of prions upon FDC in Peyer's patches and the spleen was impaired, and disease susceptibility significantly reduced. These data suggest that CXCR5-expressing conventional dendritic cells play an important role in the efficient propagation of orally administered prions toward FDC within Peyer's patches in order to establish host infection.

**IMPORTANCE** Many natural prion diseases are acquired by oral consumption of contaminated food or pasture. Once the prions reach the brain they cause extensive neurodegeneration, which ultimately leads to death. In order for the prions to efficiently spread from the gut to the brain, they first replicate upon follicular dendritic cells within intestinal Peyer's patches. How the prions are first delivered to follicular dendritic cells to establish infection was unknown. Understanding this process is important since treatments which prevent prions from infecting follicular dendritic cells can block their spread to the brain. We created mice in which mobile conventional dendritic cells were unable to migrate toward follicular dendritic cells. In these mice the early accumulation of prions on follicular dendritic cells was impaired and oral prion disease susceptibility was reduced. This suggests that prions exploit conventional dendritic cells to facilitate their initial delivery toward follicular dendritic cells to establish host infection.

## INTRODUCTION

Prion diseases, or transmissible spongiform encephalopathies (TSEs), are a unique group of subacute neurodegenerative diseases that affect humans and some domesticated and free-ranging animal species. Prion diseases are characterized by the accumulation of aggregations of PrP^Sc^, abnormally folded isoforms of the cellular prion protein (PrP^C^), in affected tissues. Prion infectivity copurifies with PrP^Sc^ and constitutes the major component of the infectious agent (1). Many prion diseases, including natural sheep scrapie, bovine spongiform encephalopathy, chronic wasting disease in cervid species, and kuru and variant Creutzfeldt-Jakob disease in humans, are acquired by peripheral exposure, such as by oral consumption of prion-contaminated food or pasture.

After ingestion many prion isolates initially accumulate and replicate first upon follicular dendritic cells (FDC) within the gut-associated lymphoid tissues (GALT) in the small intestine, such as the Peyer's patches, before they spread to the nervous system (termed neuroinvasion) (2–9). After their replication upon FDC studies in experimental rodents show that the prions subsequently infect neurons of both the sympathetic and parasympathetic nervous systems and spread along these to the central nervous system (CNS), where they ultimately cause neurodegeneration (10–12).

Mononuclear phagocytes arise from precursors in the bone marrow and comprise a heterogeneous population of monocytes, conventional dendritic cells (DC), and tissue macrophages. The different mononuclear phagocyte populations display a diverse range of roles during prion disease (13). For example, tissue macrophages appear to aid the sequestration and clearance of prions (14, 15), and microglia help protect the brain against prion-induced neuropathology (16). Conventional DC are a distinct lineage from the stromal derived FDC (17, 18) and are strategically located throughout the body to sample the local environment for pathogens and their antigens. After antigen uptake, these cells typically undergo a degree of maturation and migrate toward the draining lymphoid tissue to initiate a specific immune response. Some conventional DC populations can retain antigens in their native states and transfer them intact to naive B cells in order to initiate a specific antibody response (19). The migratory characteristics of conventional DC are exploited by some pathogens to facilitate their delivery to lymphoid tissues (20–23). Independent studies have shown that the early replication of prions upon FDC in the draining lymphoid tissue is impeded in the transient absence of conventional DC at the time of exposure (24–27). This suggests that prions may also exploit these cells to establish host infection after peripheral exposure, perhaps using them as “Trojan horses.” Whether conventional DC propagate prions toward FDC within the B cell follicles is not known. Treatments that prevent the uptake and propagation of prions to FDC can significantly delay disease pathogenesis and reduce susceptibility (24–29). A thorough understanding of the cellular mechanisms used by prions to establish infection upon FDC in the GALT may help to identify novel targets for prophylactic intervention.

Chemokines attract lymphocytes and leukocytes to lymphoid tissues and control their positioning within them. The chemokine CXCL13 is expressed by FDC and follicular stromal cells in the B-cell follicles of lymphoid tissues and mediates the homing of CXCR5-expressing cells toward them (30, 31). The migration of certain populations of conventional DC toward the FDC-containing B-cell follicles is also mediated by CXCL13-CXCR5 signaling (32–34). We therefore reasoned that if conventional DC were important for the efficient propagation of prions toward FDC in order to establish host infection, this activity would be impeded and disease susceptibility reduced in mice which specifically lacked CXCR5-expressing conventional DC. Since mice that lack Peyer's patches are refractory to oral prion infection (3, 5, 6, 35), CXCR5^−/−^ mice and CXCL13^−/−^ mice were unsuitable for use here because they also lack most Peyer's patches and certain lymph nodes (31, 36). The few Peyer's patches that do develop in these mice are much smaller, and their microarchitecture is grossly disturbed since the B cells are unable to form organized follicles (31, 36). Furthermore, as a consequence of the disturbed microarchitecture in the spleens of CXCR5^−/−^ mice, prion neuroinvasion after intraperitoneal exposure occurs at a higher rate because their FDC are abnormally superimposed over sympathetic nerves (37). Therefore, in the present study a unique compound transgenic mouse model was created in which *Cxcr5* was specifically ablated in CD11c^+^ conventional DC. These CXCR5^ΔDC^ mice were then used to test the hypothesis that conventional DC play an important role in the efficient propagation of prions toward FDC within the B cell follicles of Peyer's patches after oral exposure.

## RESULTS

### Conditional deletion of CXCR5 in CD11c^+^ cells.

To enable conditional deletion of *Cxcr5* in specific cell populations without affecting the CXCL13-CXCR5-dependent events required for normal lymphoid tissue development, mice with a conditional *Cxcr5* allele were created by introducing *loxP* sites flanking exon 2. Expression of Cre recombinase under the control of the *Itgax* locus (which encodes CD11c) in CD11c-Cre mice (38) has been used in many studies to conditionally delete the expression of target genes in conventional DC (38–40). Homozygous CXCR5^F/F^ mice were therefore crossbred to CD11c-Cre mice to generate mice specifically lacking CXCR5 expression in CD11c^+^ conventional DC, termed CXCR5^ΔDC^ mice here.

**FIG 1:**
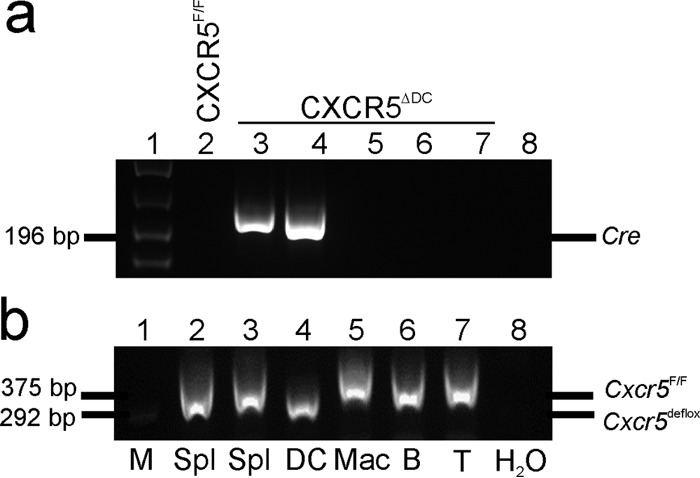
Conditional deletion of *Cxcr5* in CD11c^+^ cells. CD11c^+^ and CD11c^−^ cells were enriched from the spleens of CXCR5^ΔDC^ mice. The CD11c^−^ cells were further sorted based on their expression on CD11b, B220, and CD90.2 to broadly represent tissue macrophages, B cells, and T cells, respectively. (a) RT-PCR analysis confirmed the expression of *Cre* only in mRNA derived from splenic CD11c^+^ cells. (b) Analysis of genomic DNA confirmed that Cre recombinase-mediated recombination of the *Cxcr5* allele had only occurred in CD11c^+^ cells, as demonstrated by presence of the lower *Cxcr5*^de-flox^ band. M, DNA size markers; Spl, splenocytes; DC, CD11c^+^ conventional DC; Mac, CD11c^−^ macrophages; B, B cells; T, T cells.

CD11c^+^ and CD11c^−^ cells were enriched from the spleens of CXCR5^ΔDC^ mice. The CD11c^−^ cells were further sorted based on their expression on CD11b, B220, and CD90.2 to broadly represent tissue macrophages, B cells and T cells, respectively. Reverse transcription-PCR (RT-PCR) analysis confirmed the expression of *Cre* only in mRNA derived from splenic CD11c^+^ cells (Fig. 1a). Further PCR analysis confirmed that in CXCR5^ΔDC^ mice Cre recombinase-mediated recombination of the *Cxcr5* allele had only occurred in the genomic DNA of CD11c^+^ cells and was absent in each of the CD11c^−^ cell populations studied (Fig. 1b). These data show that in CXCR5^ΔDC^ mice, Cre recombinase-mediated recombination of *Cxcr5* is restricted to CD11c^+^ conventional DC.

### Conventional DC-specific CXCR5-deficiency does not affect secondary lymphoid tissue formation.

Next, groups of CXCR5^ΔDC^ mice and CXCR5^F/F^ (control) mice were injected intraperitoneally with Chicago Sky Blue 6B ink and analyzed 7 day later. Over the exposure period, the dye becomes concentrated within secondary lymphoid organs, enabling their macroscopic detection postmortem. The majority of the murine secondary lymphoid tissues develop consistently, whereas the lumbar aortic lymph nodes and lateral iliac lymph nodes are inconsistently present. As anticipated, the incidence and frequency of the secondary lymphoid tissues in CXCR5^F/F^ mice was equivalent to those of nontransgenic wild-type mice (41). The secondary lymphoid tissues in CXCR5^ΔDC^ mice were also present at similar incidences and frequencies to CXCR5^F/F^ control mice (Table 1), unlike those in independently generated lines of CXCR5^−/−^ mice and CXCL13^−/−^ mice (31, 36) (Table 2). These data show that a conventional DC-restricted CXCR5 deficiency does not impact lymphoid tissue organogenesis.

**TABLE 1:** Comparison of the formation and frequency of secondary lymphoid tissues in CXCR5^ΔDC^ mice and CXCR5^F/F^ (control) mice^*a*^

Secondary lymphoid tissue^*b*^	CXCR5^F/F^ mice		CXCR5^ΔDC^ mice	
	Incidence	No. present (range)	Incidence	No. present (range)
Spleen	6/6	1	8/8	1
Mandibular LN	6/6	2	8/8	2
Accessory mandibular LN	6/6	2	8/8	2
Superficial parotid LN	6/6	2	8/8	2
Cranial deep cervical LN	6/6	2	8/8	2
Proper axillary LN (brachial)	6/6	5 (4–6)	8/8	5 (4–6)
Accessory axillary LN	6/6	5 (2–7)	8/8	5 (3–6)
Subiliac LN (inguinal)	6/6	2	8/8	2
Sciatic LN	6/6	2	8/8	2
Popliteal LN	6/6	2	8/8	2
Cranial mediastinal LN	6/6	4	7/8	4
Tracheobronchial LN	6/6	1	8/8	1
Caudal mediastinal LN	6/6	1	8/8	1
Gastric LN	6/6	1	7/8	1 (0–1)
Pancreaticoduodenal LN	6/6	1	7/8	1 (0–1)
Jejunal LN (mesenteric)	6/6	5 (4–6)	8/8	5 (5–6)
Colic LN	5/6	1 (0–2)	7/8	1 (0–3)
Caudal mesenteric LN	6/6	1	8/8	1
Renal LN	6/6	2 (1–3)	8/8	2
Lumbar aortic LN*	4/6	2 (0–2)	1/8	1 (0–1)
Lateral iliac LN*	5/6	1 (0–1)	2/8	1 (0–1)
Medial iliac LN	6/6	2 (1–2)	8/8	2
External iliac LN	2/6	1 (0–1)	6/8	1 (0–1)
Peyer’s patches	6/6	6 (5–9)	8/8	6 (5–7)
Cecal patch (follicles)	6/6	5 (3–8)	7/8	2 (0–5)

a “Incidence” indicates the number of mice in which the tissue of interest was detectable/the number of mice tested; “no. present (range)” indicates the number of tissues (minimum to maximum) detectable in each mouse strain.

b LN, lymph nodes. *, Inconsistently present in wild-type mice.

**TABLE 2:** Comparison of secondary lymphoid tissue formation in CXCR5^ΔDC^ mice, CXCR5^F/F^ (control) mice, and independent lines of CXCL13^−/−^ mice and CXCR5^−/−^ mice^*a*^

Secondary lymphoid tissue	Incidence					
	Present study		Ansel et al. (31)^*b*^			
	CXCR5^F/F^ C57BL/6 cells	CXCR5^ΔDC^ C57BL/6 cells	CXCL13^+/±^ B6/129 cells	CXCL13^−/−^ B6/129 cells	CXCR5^−/−^ B6/129 cells	CXCR5^−/−^ 129 cells
Superficial parotid LN	6/6	8/8	47/47	42/46	5/5	9/10
Cranial deep cervical LN	6/6	8/8	14/16	1/14	0/5	0/10
Proper axillary (brachial) LN	6/6	8/8	42/42	1/42	2/5	0/10
Accessory axillary LN	6/6	8/8	42/42	1/42	0/5	0/10
Subiliac (inguinal) LN	6/6	8/8	41/41	0/41	0/5	0/10
Popliteal LN	6/6	8/8	15/19	0/18	0/5	10/10
Jejunal (mesenteric) LN	6/6	8/8	50/50	50/50	5/5	10/10
Renal LN	6/6	8/8	12/15	0/12	0/5	3/9
Medial iliac LN	6/6	8/8	38/38	0/41	0/5	0/10
Peyer’s patches^*c*^	6/6 (6, 5–9)	8/8 (6, 5–7)	31/31 (2 × 3–6 29 × > 6)	16/37 (21 × 0, 11 × 1–2, 5 × 3–6)	4/5 (1 × 0, 2 × 1–2, 1 × 3–6, 1 × > 6)	9/9 (5 × 1–2, 3 × 3–6, 1 × > 6)

a The mouse strain background is specified in each column subheading. “Incidence” is expressed as the number of mice in which the tissue of interest was detectable/the number of mice tested. LN, lymph nodes.

b Data are derived from a study by Ansel et al. (31).

c The “median number of Peyer′s patches/mouse, range” is indicated within parentheses for the present study. For the study by Ansel et al., the values indicate “*n* × 3–6”, where *n* is the number of mice with Peyer’s patches within the ranges indicated.

### Conventional DC from CXCR5^ΔDC^ mice have impaired chemotaxis toward CXCL13.

**FIG 2:**
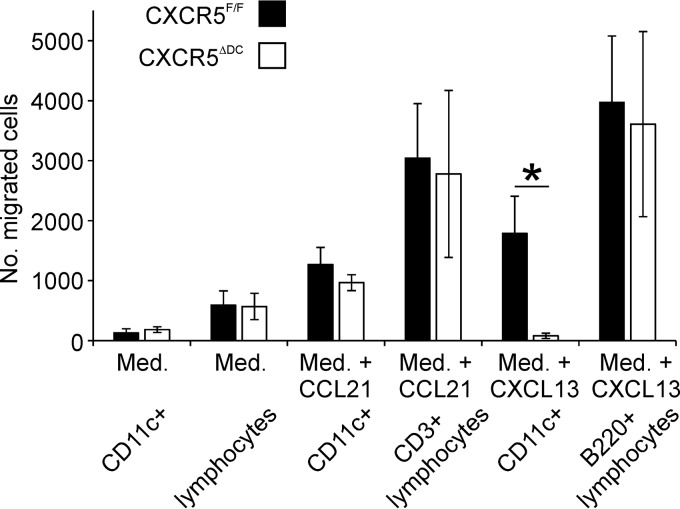
CD11c^+^ conventional DC from CXCR5^ΔDC^ mice have impaired chemotaxis toward CXCL13. *Ex vivo* chemotaxis of mesenteric lymph node (MLN) cells from CXCR5^ΔDC^ mice and CXCR5^F/F^ control mice toward CXCL13 or CCL21 (200 ng/ml). The number of CD11c^+^ cells, CD3^+^ lymphocytes (T cells) and B220^+^ lymphocytes (B cells) which had migrated into the lower chamber after 24 h was determined by flow cytometry. Med., medium alone was used as a control. The ability of CD11c^+^ conventional DC from CXCR5^ΔDC^ mice to migrate toward CXCL13 was significantly impeded. *, *P* < 0.024 (*n* = 5 wells/treatment).

The chemokine CXCL13 is expressed by FDC and follicular stromal cells in the B-cell follicles of lymphoid tissues and mediates the homing of CXCR5-expressing cells toward them (30, 31). *Ex vivo* chemotaxis assays confirmed that the migration of CD11c^+^ conventional DC from CXCR5^ΔDC^ mice toward CXCL13 was significantly impeded compared to conventional DC from CXCR5^F/F^ control mice (Fig. 2; *P* < 0.024). In contrast, the chemotaxis of B cells (B220^+^ cells) from CXCR5^ΔDC^ mice toward CXCL13 was equivalent to that observed from cells from CXCR5^F/F^ mice. The ability of conventional DC to migrate toward the chemokine CCL21 (which signals via CCR7) was also similar in cells from each mouse group.

### Lymphoid tissue microarchitecture in CXCR5^ΔDC^ mice.

**FIG 3:**
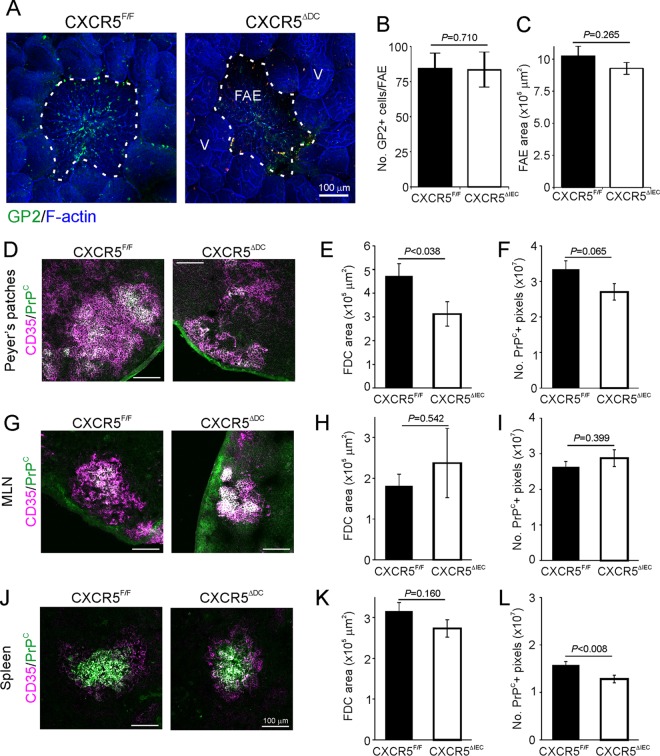
Comparison of M cell and follicular dendritic cell (FDC) status in the secondary lymphoid tissues of CXCR5^ΔDC^ mice and CXCR5^F/F^ control mice. (A) Peyer's patches were whole-mount immunostained to detect M cells (GP2^+^ cells, green). F-actin (blue) was used as a counterstain. The broken line indicates the boundary of the follicle associated epithelium (FAE). V, villi. (B) The number of GP2^+^ M cells/FAE was similar in Peyer's patches from each mouse group (*P* = 0.710). (C) Morphometric analysis suggested the size of the FAE area was also similar in each mouse group (*P* = 0.265 [Student *t* test]; data were derived from 2 to 20 FAE/mouse [*n* = 6 to 8 mice/group]). IHC comparisons of CD35 (magenta) and PrP^C^ (green) expression by FDC in the Peyer's patches (D), mesenteric lymph nodes (MLN) (G), and spleens (J) of CXCR5^F/F^ and CXCR5^ΔDC^ mice are shown. The sizes of the FDC in the Peyer's patches (E), MLN (H), and (K) spleens of mice from each group were estimated by morphometric analysis of the area of CD35^+^ immunostaining. The abundance of PrP^C^ expressed by the FDC in Peyer's patches (F), MLN (I), or spleens (L) from each mouse group was estimated by morphometric analysis of the number of PrP^C+^ pixels within each FDC network. Data were derived from 2 to 10 FDC/mouse (*n* = 6 to 8 mice/group).

The sampling of prions across the intestinal epithelium by M cells, and their subsequent early replication upon PrP^C^-expressing FDC is obligatory for efficient neuroinvasion after oral exposure (4–6, 29, 42). We therefore determined whether the development of these cells was affected in the lymphoid tissues of CXCR5^ΔDC^ mice. Whole-mount immunostaining using the mature M-cell marker glycoprotein 2 (GP2 [43, 44]) revealed similar densities of GP2^+^ M cells in the follicle-associated epithelia (FAE) overlying the Peyer's patches and CXCR5^ΔDC^ mice and CXCR5^F/F^ control mice (Fig. 3A and B). The size of the FAE was also equivalent (Fig. 3C). Unlike the disturbed distribution of FDC in the spleens of CXCL13^−/−^ mice (37), those in the lymphoid tissues of CXCR5^ΔDC^ mice formed distinct networks. Furthermore, immunohistochemistry (IHC) and morphometric analysis suggested that FDC size (the area occupied by CD35^+^ immunostaining) was similar in the mesenteric lymph nodes (MLN) and spleens from CXCR5^ΔDC^ and CXCR5^F/F^ mice (Fig. 3H and K, respectively), although those in the Peyer's patches of CXCR5^ΔDC^ mice were slightly smaller (Fig. 3E). However, the abundance of PrP^C+^ immunostaining upon FDC in Peyer's patches and MLN from each mouse group was similar (Fig. 3F and I, respectively), although a marginal reduction was in observed in the spleens of CXCR5^ΔDC^ mice (Fig. 3L).

### Prion accumulation in lymphoid tissues is reduced in orally exposed CXCR5^ΔDC^ mice.

In order to determine the effects of conventional DC-specific CXCR5-deficiency on oral prion pathogenesis, groups of CXCR5^ΔDC^ mice and CXCR5^F/F^ mice were orally exposed to ME7 scrapie prions and Peyer's patches, MLN, and spleens collected at intervals afterwards (*n* = 4/group). Prion disease-specific, abnormal accumulations of PrP (referred to as PrP^d^) characteristically present only in prion-affected tissues were detected by IHC (4–6, 29, 45–48). Paraffin-embedded tissue (PET) blot analysis of adjacent membrane-bound sections was used to confirm that these PrP^d^ aggregates contained prion disease-specific, relatively proteinase K (PK)-resistant PrP^Sc^ (49).

**FIG 4:**
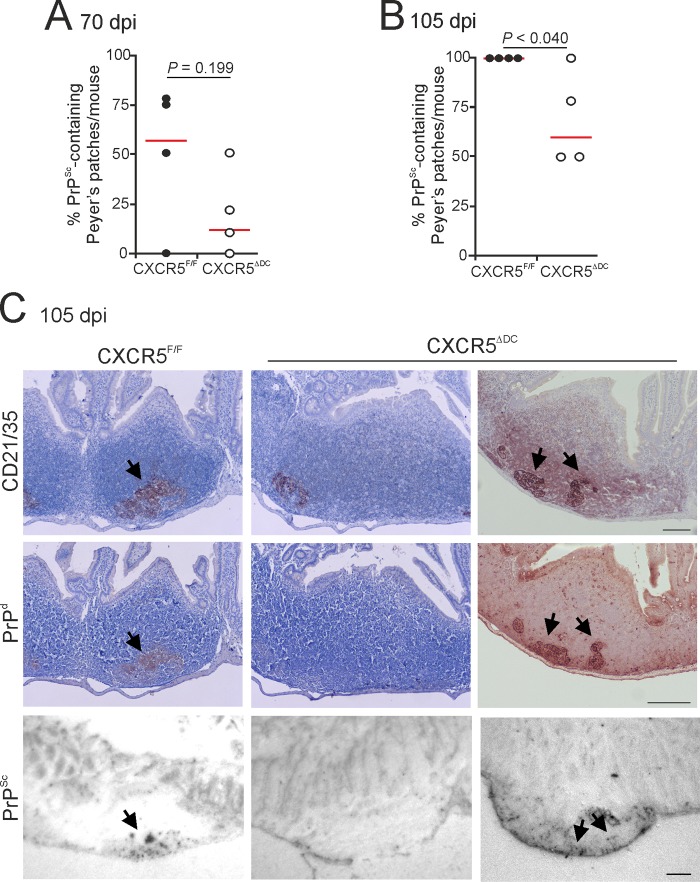
The early accumulation of prions in the Peyer's patches is delayed in CXCR5^ΔDC^ mice. Mice were orally exposed to ME7 scrapie prions; Peyer's patches were collected at 70 and 105 dpi and analyzed by IHC and PET immunoblot analysis. At 70 (A) and 105 (B) dpi, the frequencies of FDC networks containing PrP^Sc^ in the Peyer's patches from each mouse group were compared (*n* = 4 mice/group). Horizontal bar, median. (C) IHC analysis showed that at 105 dpi, high levels of disease-specific PrP (PrP^d^; brown, middle row, arrows) were detected in association with FDC (CD21/35^+^ cells; brown, upper row) in Peyer's patches from CXCR5^F/F^ control mice. Sections were counterstained with hematoxylin to detect cell nuclei (blue). Analysis of adjacent sections by PET immunoblot analysis confirmed the presence of prion-specific PK-resistant PrP^Sc^ (blue/black). In contrast, although PrP^Sc^ was detectable in association with FDC in some of the tissues from orally exposed CXCR5^ΔDC^ mice (right-hand column), many FDC in the Peyer's patches of CXCR5^ΔDC^ mice lacked PrP^Sc^ accumulation (middle column). Scale bar, 100 μm.

As anticipated, by 70 days postinfection (dpi), abundant PrP^Sc^ accumulations were detected in association with FDC (CD21/35^+^ cells) in the majority of the Peyer's patches from CXCR5^F/F^ control mice (Fig. 4A). By 105 dpi, heavy FDC-associated PrP^Sc^ accumulations were detected in all the Peyer's patches of CXCR5^F/F^ mice (Fig. 4B and C). However, the incidence of the PrP^Sc^ accumulation in Peyer's patches of CXCR5^ΔDC^ mice was reduced at each of these times after prion exposure (Fig. 4A to C).

**FIG 5:**
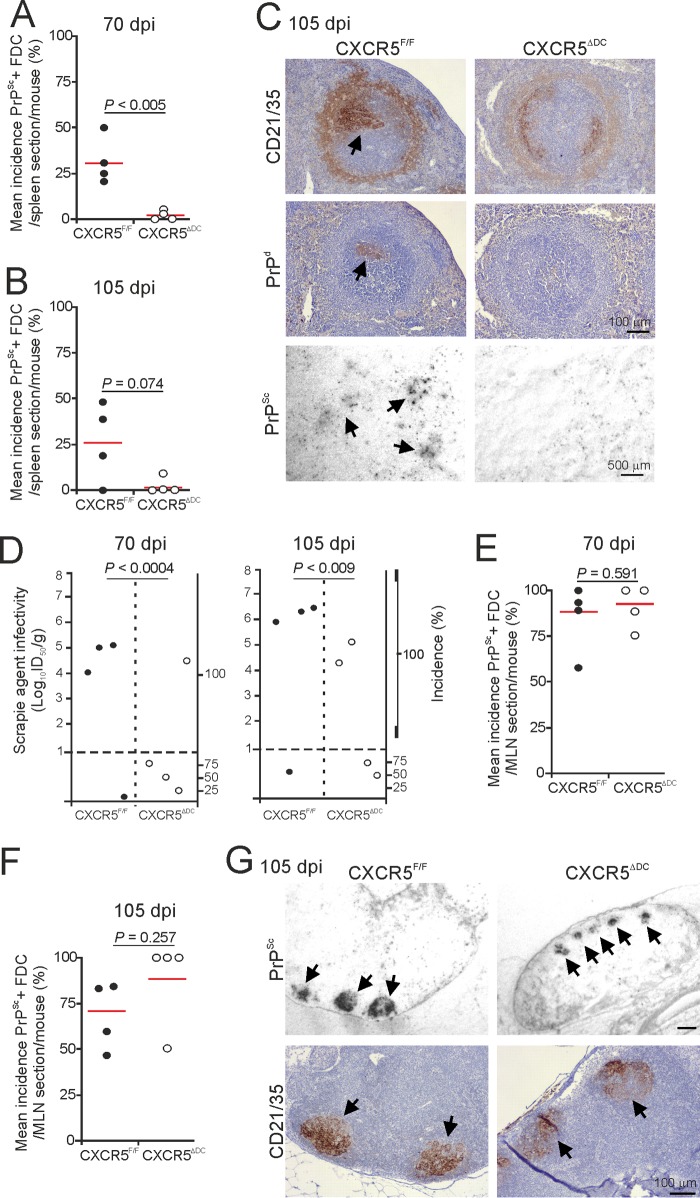
Comparison of prion accumulation in the spleens and MLN of orally exposed CXCR5^F/F^ and CXCR5^ΔDC^ mice. Mice were orally exposed to ME7 scrapie prions, and spleens and MLN were collected at 70 and 105 dpi. At 70 (A) and 105 (B) dpi, the frequencies of FDC networks containing PrP^Sc^ in the spleens from CXCR5^ΔDC^ mice were reduced compared to CXCR5^F/F^ control mice (*n* = 4 mice/group). Horizontal bar, median. (C) IHC analysis showed that at 105 dpi high levels of disease-specific PrP (PrP^d^; brown, middle row, arrow) were detected in association with FDC (CD21/35^+^ cells; brown, upper row) in spleens from CXCR5^F/F^ control mice. Sections were counterstained with hematoxylin to detect cell nuclei (blue). Analysis of adjacent sections by PET immunoblot analysis confirmed the presence of prion-specific PK-resistant PrP^Sc^ (blue/black). In contrast, PrP^Sc^ was not detectable in association with the majority of the FDC in spleens from orally exposed CXCR5^ΔDC^ mice. (D) Prion infectivity levels were assayed in spleens from CXCR5^F/F^ and CXCR5^ΔDC^ mice (*n* = 4 spleens/group) collected at 70 and 105 dpi. Prion infectivity titers (log_10_ ID_50_/g tissue) were determined by the injection of tissue homogenates into groups of tga20 indicator mice (*n* = 4 recipient mice/spleen). Each symbol represents data derived from an individual spleen. Data below the horizontal line indicate disease incidence in the recipient mice <100% and considered to contain trace levels of prion infectivity. IHC and PET blot analysis suggested that the frequencies of FDC networks containing PrP^Sc^ in the MLN of CXCR5^ΔDC^ and CXCR5^F/F^ control mice were similar at 70 (E) and 105 (F) dpi (*n* = 4 mice/group). (G) Analysis of adjacent sections showed that at 105 dpi high levels of PrP^Sc^ (blue/black, upper row, arrows) were detected in association with FDC (CD21/35^+^ cells; brown, low row) in MLN from each mouse group. Sections were counterstained with hematoxylin to detect cell nuclei (blue).

Within weeks of oral exposure, high levels of ME7 scrapie prions first accumulate upon FDC in the Peyer's patches and are subsequently disseminated via the blood and lymph to most other lymphoid tissues (4–6, 24, 29, 50). In the spleens of orally exposed CXCR5^F/F^ control mice, some FDC-associated PrP^Sc^ accumulations were first evident at 70 dpi (Fig. 5A to C). However, the abundance of these FDC-associated PrP^Sc^ accumulations was reduced in the spleens of CXCR5^ΔDC^ mice at 70 and 105 dpi (Fig. 5A to C). To compare the prion infectivity levels within these tissues, spleen homogenates were prepared and injected intracerebrally (i.c.) into groups of tga20 indicator mice (*n* = 4 recipient tga20 mice/spleen homogenate). High levels of prion infectivity were detected in the majority of the spleens collected from the CXCR5^F/F^ control mice at 70 dpi and had increased in magnitude by 105 dpi (Fig. 5D). In contrast, significantly lower levels of prion infectivity were detected in the spleens of orally exposed CXCR5^ΔDC^ mice (Fig. 5D; 70 dpi, *P* < 0.0004; 105 dpi, *P* < 0.009; log-rank [Mantel-Cox] test). Only trace levels of prion infectivity were detected in three of four spleens from the CXCR5^ΔDC^ mice analyzed at 70 dpi and in two of the four spleens analyzed at 105 dpi.

Together, these data show that in the specific absence of CXCR5-expressing conventional DC at the time of oral exposure, the early accumulation of prions upon FDC in the Peyer's patches and spleen is significantly delayed. However, abundant levels of PrP^Sc^ were evident in association with FDC in the MLN of CXCR5^ΔDC^ and CXCR5^F/F^ mice at each time point after oral exposure to ME7 scrapie prions (Fig. 5E to G). This implied that the accumulation of prions upon FDC in the MLN was unaffected by an absence of CXCR5-expressing conventional DC.

### Oral prion disease susceptibility is reduced in the specific absence of CXCR5-expressing conventional DC.

**FIG 6:**
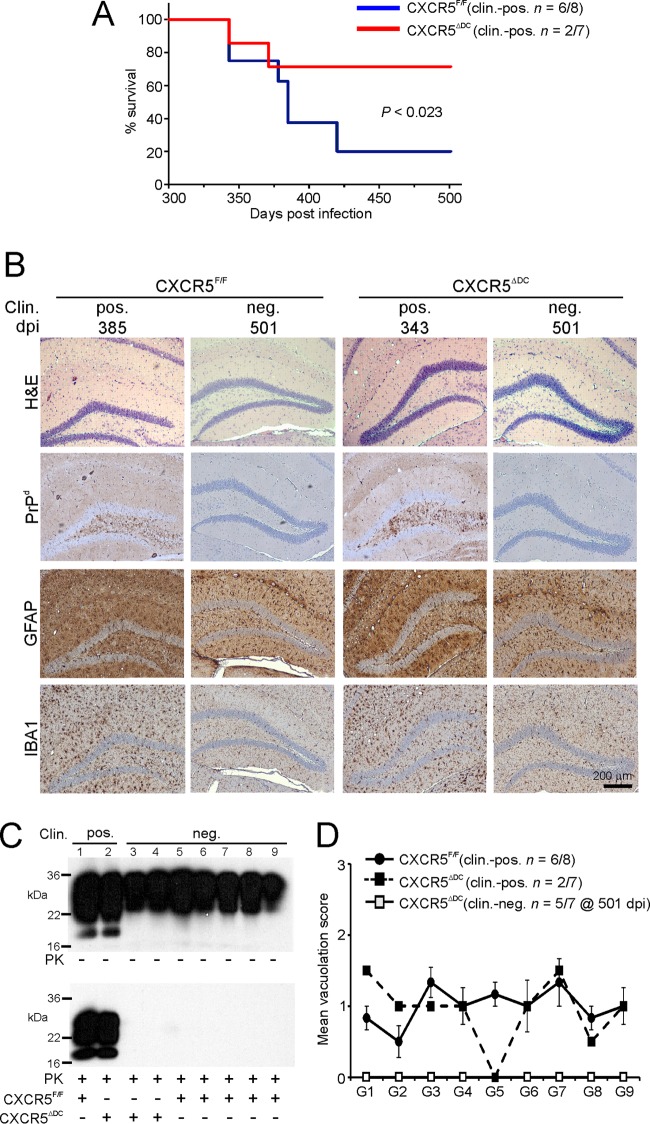
Oral prion disease susceptibility is reduced in CXCR5^ΔDC^ mice. (A) Disease susceptibility after oral exposure of CXCR5^ΔDC^ (red) and CXCR5^F/F^ control (blue) mice to a limiting dose of prions (*P* < 0.023 [Fisher exact test]. (B) High levels of spongiform pathology (hematoxylin and eosin staining [H&E]), heavy accumulations of disease-specific PrP, (PrP^d^; brown), reactive astrocytes expressing GFAP (brown), and active microglia expressing IBA1 (brown) were detected in the brains of all orally exposed mice with clinical prion disease. None of these histopathological signs of prion disease were detected in the brains of any of the clinically negative mice up to at least 501 days after oral exposure. Clin., clinical prion disease status; pos., clinically positive; neg., clinically negative; individual survival times are shown. Sections were counterstained with hematoxylin to detect cell nuclei (blue). (C) Immunoblot analysis of brain tissue homogenates confirmed the presence of high levels of prion-specific, relatively PK-resistant PrP^Sc^ within the brains of the clinically affected mice from each group. However, no PrP^Sc^ was detected in the brains of any of the clinically negative CXCR5^ΔDC^ mice. Samples were treated in the presence (lower panel) or absence (upper panel) of PK before electrophoresis. After PK treatment, a typical three-band pattern was observed between molecular mass value of 20 to 30 kDa, representing unglycosylated, monoglycosylated, and diglycosylated isomers of PrP (in order of increasing molecular mass). (D) The severity and distribution of the spongiform pathology (vacuolation) within each brain was scored on a scale of 1 to 5 in nine gray-matter areas: G1, dorsal medulla; G2, cerebellar cortex; G3, superior colliculus; G4, hypothalamus; G5, thalamus; G6, hippocampus; G7, septum; G8, retrosplenial and adjacent motor cortex; and G9, cingulate and adjacent motor cortex. Each point represents the mean vacuolation score ± SE.

Since oral exposure to a limiting dose of ME7 scrapie prions typically yields a disease incidence of <100% in wild-type (control) mice, it was used here to enable the effects of conventional DC-restricted CXCR5-deficiency on survival time and prion disease susceptibility to be determined. As anticipated, the majority of the orally exposed CXCR5^F/F^ (control) mice succumbed to clinical prion disease (Fig. 6A; mean, 376 ± 12 days; median, 382 days; *n* = 6/8). In contrast, disease susceptibility was significantly reduced in the orally exposed CXCR5^ΔDC^ mice, since only two of seven mice succumbed to clinical prion disease with individual survival times of 343 and 371 days: the remaining five mice did not develop clinical signs of prion disease up to at least 501 dpi (Fig. 6A; *P* < 0.023 [Fisher exact test]). All of the brains from the mice in each group that developed clinical signs of prion disease had the characteristic spongiform pathology (vacuolation), disease-specific PrP accumulation, astrogliosis, and microgliosis and typically associated with terminal infection with ME7 scrapie prions (Fig. 6B and C). The distribution and severity of the spongiform pathology was also similar in the brains of all the clinically affected mice (Fig. 6D), indicating that conventional DC-specific CXCR5 deficiency did not alter the course of CNS prion disease once neuroinvasion had occurred. No histopathological signs of prion disease were detected in the brains of any of the clinically negative mice.

**FIG 7:**
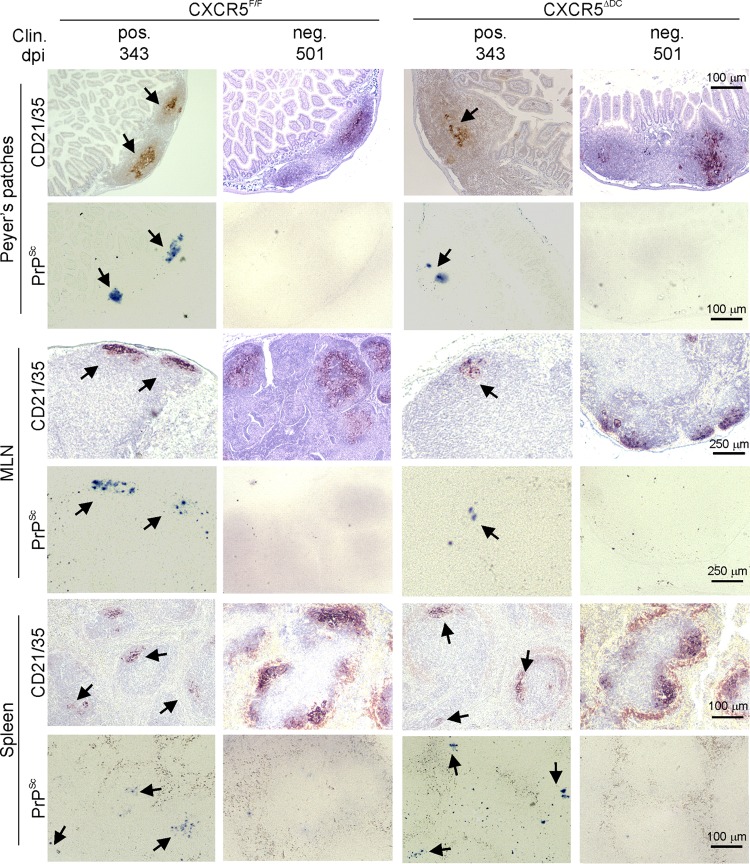
Prion accumulation in the lymphoid tissues of CXCR5^ΔDC^ and CXCR5^F/F^ mice at the terminal stage of disease. Mice were orally exposed to ME7 scrapie prions, and Peyer's patches, MLN, and spleens were collected from all clinically affected mice and from those free of clinical signs of prion disease at the end of the experiment at 501 dpi. Clin., clinical prion disease status; pos., clinically positive; neg. clinically negative. Individual survival times are shown. High levels of PrP^Sc^ (PET immunoblot; black, arrows) were detected in association with follicular dendritic cells (CD21/35^+^ cells; brown, arrows) in the Peyer's patches, MLN, and spleens from all clinically affected mice. In contrast, no PrP^Sc^ was detected in tissues from any of the clinically negative survivors at 501 dpi. Sections were counterstained with hematoxylin to detect cell nuclei (blue). CXCR5^F/F^ Clin. pos, *n* = 6 mice; CXCR5^F/F^ Clin. neg, *n* = 2 mice; CXCR5^ΔDC^ Clin. pos, *n* = 2 mice; CXCR5^ΔDC^ Clin. neg, *n* = 5 mice.

After oral exposure, high levels of ME7 scrapie prions are maintained upon FDC in lymphoid tissues until the terminal stages of disease (5, 6, 24, 29). Here, FDC-associated PrP^Sc^ accumulations were detected in the Peyer's patches, MLN, and spleens of all clinically affected CXCR5^ΔDC^ and CXCR5^F/F^ mice (Fig. 7). In comparison, no evidence of PrP^Sc^ accumulation was observed in any of the tissues from the clinically negative mice (Fig. 7). These data indicate that all of the clinically negative mice were free of prions in their lymphoid tissues and brains and were highly unlikely to succumb clinical prion disease after substantially extended survival times. Together, these data show that CXCR5-expressing conventional DC are essential for efficient prion neuroinvasion after oral exposure.

### Conventional DC-specific CXCR5 deficiency does not influence prion disease susceptibility when infection is established directly within the CNS.

**FIG 8:**
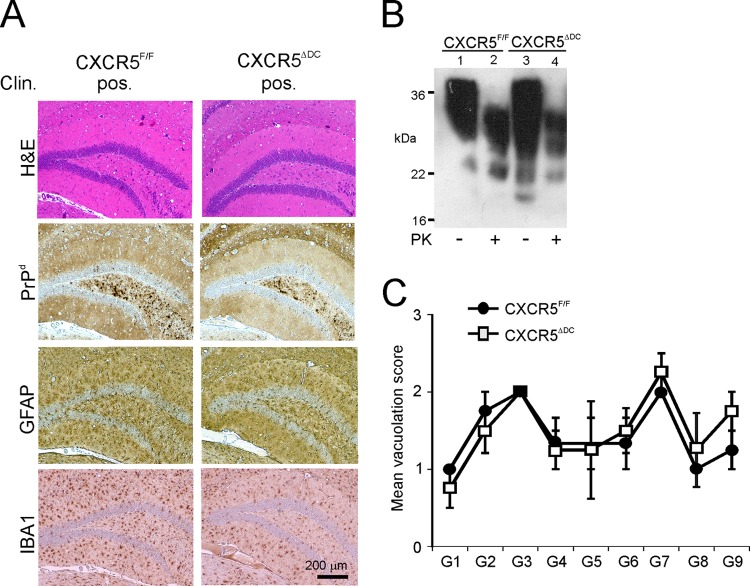
Neuropathological comparison of brains from clinically affected CXCR5^ΔDC^ and CXCR5^F/F^ mice after injection of prion directly into the CNS. CXCR5^ΔDC^ and CXCR5^F/F^ mice (*n* = 4/group) were injected i.c. with ME7 scrapie prions, and brains were collected at the terminal stage of disease. (A) Histopathological analysis revealed the typical spongiform pathology (H&E, top row), heavy accumulations of prion disease-specific PrP^d^ (brown, second row), reactive astrocytes expressing GFAP (brown, third row), and active microglia expressing IBA1 (brown, bottom row) in the brains of all clinically affected mice from each group. Sections were counterstained with hematoxylin to detect cell nuclei (blue). (B) Immunoblot analysis of brain tissue homogenates confirmed the presence of high levels of prion-specific, relatively PK-resistant PrP^Sc^ within the brains of mice from each group. Samples were treated in the presence (+) or absence (−) of PK before electrophoresis. After PK treatment, a typical three-band pattern was observed between molecular mass values of 20 to 30 kDa, representing unglycosylated, monoglycosylated, and diglycosylated isomers of PrP (in order of increasing molecular mass). (C) The severity and distribution of the spongiform pathology (vacuolation) within the brains of all clinically affected CXCR5^ΔDC^ and CXCR5^F/F^ mice was similar. The severity of the vacuolation in each brain was scored on a scale of 1 to 5 in the following gray matter regions: G1, dorsal medulla; G2, cerebellar cortex; G3, superior colliculus; G4, hypothalamus; G5, thalamus; G6, hippocampus; G7, septum; G8, retrosplenial and adjacent motor cortex; and G9, cingulate and adjacent motor cortex. Each point represents the mean vacuolation score ± the SE.

When groups of CXCR5^ΔDC^ and CXCR5^F/F^ mice were injected i.c. with ME7 scrapie prions directly into the CNS, all mice succumbed to clinical disease with similar survival times (CXCR5^ΔDC^ mice, 157 ± 2 days, *n* = 4/4; CXCR5^fl^ mice, 162 ± 3 days, *n* = 4/4; *P* = 0.302). Histopathological analysis of the brains from each group of clinically affected mice revealed the characteristic neuropathology and PrP^Sc^ accumulation associated with terminal infection with ME7 scrapie prions (Fig. 8A and B). The distribution and severity of the spongiform pathology was also similar in the brains of the clinically affected CXCR5^ΔDC^ and CXCR5^F/F^ mice (Fig. 8C). Therefore, a CXCR5 deficiency specifically in conventional DC did not affect prion disease pathogenesis or susceptibility when the infection was established directly within the CNS. These data are consistent with the independent observation that prion propagation within the CNS is not affected in CXCR5^−/−^ mice (37).

### Splenectomy before oral prion exposure does not influence disease susceptibility.

**FIG 9:**
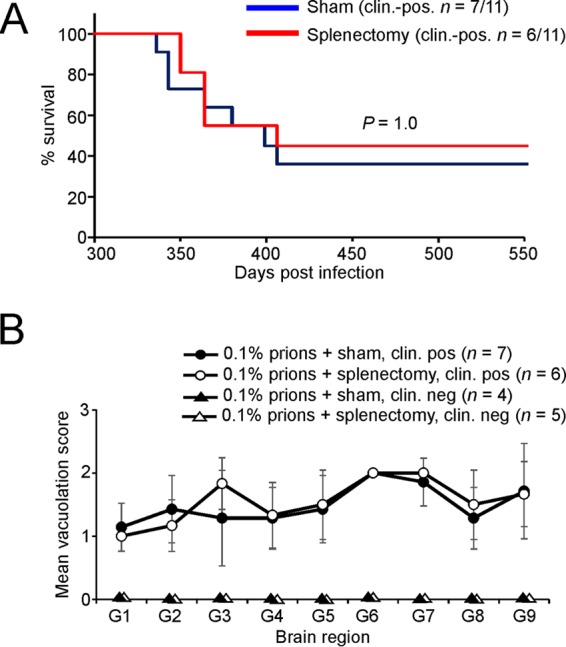
Splenectomy before oral prion exposure does not influence disease susceptibility. C57BL/Dk wild-type mice were surgically splenectomized, or sham operated (as a control), and 8 days later orally exposed to a 0.1% dose of ME7 scrapie prions (*n* = 11/group). (A) Comparison of disease susceptibility of splenectomized (red) and sham-operated mice (blue) to oral prion infection (*P* = 1.0 [Fisher exact test]). (B) The severity and distribution of the spongiform pathology (vacuolation) within the brains of all clinically affected splenectomized (open circles) and sham-operated mice (closed circles) was similar. The severity of the vacuolation in each brain was scored on a scale of 1 to 5 in the following gray-matter regions: G1, dorsal medulla; G2, cerebellar cortex; G3, superior colliculus; G4, hypothalamus; G5, thalamus; G6, hippocampus; G7, septum; G8, retrosplenial and adjacent motor cortex; and G9, cingulate and adjacent motor cortex. Each point represents the mean vacuolation score ± the SE.

The data presented above show that the early accumulation of prions in the Peyer's patches (Fig. 4) and spleens (Fig. 5) of orally exposed CXCR5^ΔDC^ mice was impaired. Although early prion replication upon FDC in the Peyer's patches in the small intestine is essential for efficient neuroinvasion after oral exposure (5, 6, 45), we considered it plausible that the effects observed here on disease susceptibility were due to impaired neuroinvasion from the spleen. If the spleen did play an important role in prion neuroinvasion after oral exposure, we reasoned that splenectomy before prion exposure would similarly impede neuroinvasion and reduce disease susceptibility. To test this hypothesis, wild-type mice were surgically splenectomized and 8 days later orally exposed to a 0.1% dose of ME7 scrapie prions as described above. A parallel group of mice were sham operated before oral prion exposure as a control. Our data show that splenectomy did not influence prion disease pathogenesis or susceptibility as mice from each treatment group succumbed to clinical disease with similar survival times and disease incidences (Fig. 9A; splenectomized mice, mean = 371 ± 13 days, median = 364 days, *n* = 6/11; sham-operated mice, mean = 367 ± 11 days, median = 364 days, *n* = 7/11 [*P* = 1.0, Fisher exact test]). The distribution and severity of the spongiform pathology were also similar in the brains of all the clinically affected splenectomized and sham-operated mice (Fig. 9B). These data clearly show that the spleen does not play an important role in neuroinvasion after oral prion exposure. This suggests that the major effects observed on oral prion disease pathogenesis and susceptibility in CXCR5^ΔDC^ mice were not due to impaired neuroinvasion from their spleens.

## DISCUSSION

The early replication of prions upon FDC in Peyer's patches is essential for efficient neuroinvasion from the small intestine after oral exposure (3–6, 35). The cellular mechanism by which orally acquired prions are initially conveyed to FDC in order to establish host infection was uncertain. Production of the chemokine CXCL13 by FDC and follicular stromal cells plays an important role in attracting CXCR5-expressing cells toward the FDC-containing B-cell follicles of secondary lymphoid organs (31, 36, 51). Here, a unique compound transgenic mouse model was created in which CXCR5 deficiency was restricted to CD11c^+^ conventional DC. In the specific absence of CXCR5-expressing conventional DC in CXCR5^ΔDC^ mice, the early accumulation of prions upon FDC in the Peyer's patches and spleen was reduced, significantly reducing disease susceptibility. Our data suggest that CXCR5-expressing conventional DC are required for the efficient delivery of orally acquired prions to FDC in the Peyer's patches of the small intestine in order to achieve host infection.

The data presented here help advance our understanding of how orally acquired prions utilize an elegant cellular relay to establish host infection from the lumen of the small intestine. Prions are first transported across the gut epithelium by M cells in the FAE overlying the Peyer's patches (29, 45, 52, 53). This initial transport of prions across the gut epithelium by M cells is essential to establish infection in Peyer's patches (29, 45). Antigens and particles that have been transported across the gut epithelium by M cells are released into their underlying basolateral pockets where they are sampled by mononuclear phagocytes (54). Our data suggest that prions are subsequently acquired by conventional DC (24, 55) and propagated by them in a CXCL13-CXCR5-dependent manner toward FDC within the B-cell follicles of Peyer's patches. The prions are then acquired by FDC and amplified upon their surfaces above the threshold required to achieve neuroinvasion (4–6, 42, 55). Studies from experimental mice indicate that prions can establish infection in enteric nerves within 21 days after oral exposure (4, 55). How the prions spread between the FDC and enteric nerves is not known, but a role for conventional DC in this process has been proposed (56–58).

Whether these apparent prion propagation activities are restricted to certain conventional DC populations is uncertain, but the specific depletion of CD8^+^ CD11c^+^ cells does not influence oral prion disease pathogenesis, implying that CD8^+^ conventional DC do not play a role (26). Indeed, within Peyer's patches CD8^+^ conventional DC are rarely encountered within the subepithelial dome region immediately beneath the M-cell-containing FAE. Instead, these cells are mostly localized within the T-cell-rich interfollicular regions (59). Splenic plasmacytoid DC also accumulate high levels of infectious prions during infection (60) but are unlikely to play a significant role in prion propagation since they do not migrate in the lymphatics during the steady state or after activation (61).

Whether the initial uptake of prions by conventional DC involves a specific receptor is uncertain. Most mononuclear phagocyte populations express cellular PrP^C^ (62–64), but the propagation of prions from peripheral exposure sites to FDC is not affected by the absence of PrP^C^ expression in hematopoietic cells (42, 48, 65–67). Conventional DC, like the FDC in the B-cell follicles (28, 68–71), can acquire prions after their opsonization by complement components such as C1q and C3 (72, 73). Depending on their location, subset, and activation status, certain populations of conventional DC express a variety of complement receptors (CR), including CR1 (CD35), CR2 (CD21), CR4 (CD11c/CD18), calreticulin, CD93, and SIGN-R1 (CD209b), but it is uncertain whether they also facilitate the complement-mediated uptake of prions by these cells (72, 73). However, SIGN-R1 is unlikely to play a role since its transient downregulation before prion exposure does not influence disease pathogenesis (74). The possibility also cannot be excluded that conventional DC simply acquire prions nonspecifically as they constitutively sample their microenvironments via fluid-phase micropinocytosis.

Although the data in the present study suggest that CXCR5-expressing conventional DC are required for the efficient propagation of prions to FDC in Peyer's patches, it is not known how they are transferred between these cell populations. Follicular B cells within the subcapsular sinus of lymph nodes acquire lymph-borne immune complexes via their CR and deliver them to FDC. The higher immune-complex-binding affinity of FDC appears to enable them to strip the B cells of their immune-complex cargo as they migrate into the follicles (75–77). Conventional DC can retain PrP^Sc^ on both the cell surface and in intracellular compartments (73). Since the expression level of CR1 and CR2 on FDC is greater than the surrounding lymphocytes and leukocytes, it is plausible that complement-opsonized prions are stripped from the surfaces of conventional DC by FDC in a similar manner. Alternatively, it is plausible that conventional DC might indirectly deliver infectious prions toward FDC. Since CR-expressing marginal zone B cells in the spleen can shuttle complement-opsonized antigens to the FDC (78), B cells may similarly strip opsonized prions from conventional DC in the vicinity of the B cell follicle and deliver them to the FDC.

Tunneling nanotubes (TNT) are thin, membrane-bound cylinders of cytoplasm that can connect neighboring cells to enable cell-to-cell communication and the intercellular transfer of plasma membrane or cytoplasmic components. These TNT structures are exploited by HIV-1 as a means of intercellular transfer between T cells (79) and to shuttle virus-encoded immunosuppressive factors from infected macrophages to B cells to suppress the humoral response (80). *In vitro* coculture studies suggest prions may also transfer between conventional DC to neurons via endolysosomal vesicles within TNT (57, 58, 81, 82). Infectious prions may also be released from infected cells in the form of small endosome-derived vesicles termed exosomes (83) and, in doing so, enhance their ability to infect neighboring cells (60). Whether the transfer of prions between conventional DC and FDC occurs *in vivo* via one or a combination of the above processes remains to be determined.

We have previously shown that although the transient depletion of CD11c^+^ cells impedes the accumulation of prions in Peyer's patches and reduces disease susceptibility, a small number of mice did develop clinical disease (24). In the present study, we observed a similar effect on prion disease pathogenesis in the absence of CXCR5-expressing conventional DC: prion accumulation was impeded in the Peyer's patches and spleens of orally exposed CXCR5^ΔDC^ mice, and disease susceptibility was reduced, but a small number of mice eventually succumbed to clinical prion disease. These studies suggest that conventional DC provide an efficient route by which prions are initially conveyed to FDC. However, in their absence a limited amount of prions are able to avoid clearance by cells such as tissues macrophages (14, 15) and reach the FDC via alternative and less efficient routes (27, 73, 84).

Studies in experimental mice show that after oral exposure, prions accumulate first in the Peyer's patches in the small intestine and then spread via the blood and lymph to most other secondary lymphoid tissues, including the spleen (4–6, 24, 50). Analysis of the pathogenesis of natural sheep scrapie suggests a similar temporal distribution (9). In the present study, the early accumulation of prions within the MLN of CXCR5^ΔDC^ mice was not impaired, suggesting that the prions had established infection within the MLN independently of CXCR5-expressing conventional DC. Although prions also replicate upon FDC in the MLN soon after oral exposure, our studies in mice have shown that the MLN do not influence prion neuroinvasion from the intestine (5, 6, 45).

We also showed that early prion replication in the spleens of CXCR5^ΔDC^ mice was significantly reduced. This raised the possibility that the significantly reduced prion disease susceptibility observed in these mice was, at least in part, due to impaired neuroinvasion from their spleens. However, splenectomy of conventional mice prior to oral exposure with ME7 scrapie prions did not influence survival times or disease susceptibility. These data are consistent with an earlier study using mouse-passaged 139A scrapie prions which also suggested that the spleen does not play a role in prion disease pathogenesis after intragastric exposure (85), and our subsequent demonstrations that the GALT in the small intestine are the important sites of early prion accumulation and neuroinvasion after oral exposure (5, 6).

Many studies have defined conventional DC based on their expression of distinct cell surface markers such as the integrin CD11c. Murine conventional DC express CD11c highly, and this has been used as a reliable marker for these cells in a wide range of studies. However, the expression of this integrin is not restricted to conventional DC since certain macrophage populations and activated monocytes can also express CD11c to some degree (86, 87). The recent discovery that expression of the zinc finger transcription factor ZBTB46 is restricted to conventional DC (88, 89) provides an excellent opportunity to create conditional knockout mouse models to further define the separate roles of conventional DC and tissue macrophages in prion disease pathogenesis (90).

In conclusion, our data demonstrate that prions exploit conventional DC in order to facilitate their efficient propagation to FDC in Peyer's patches in order to establish host infection. The status of conventional DC is dramatically influenced by microbial stimuli and inflammatory conditions in the intestine and can enhance the uptake of certain pathogens from the gut lumen (91–93). Treatments that prevent the initial uptake and accumulation of prions in Peyer's patches can significantly reduce disease susceptibility (4, 24, 29). Therefore, a thorough understanding of the cellular and molecular mechanisms which prions exploit to establish infection upon FDC in the GALT may help to identify important factors that influence disease susceptibility or novel targets for prophylactic intervention.

## MATERIALS AND METHODS

### Mice.

The following mouse strains were used in this study where indicated: CD11c-Cre (38); and tga20, which overexpress PrP^C^ (94). *Cxcr5*^F/F^ mice were produced by Ozgene (Bentley DC, Australia) and created by introducing *loxP* sites flanking exon 2 of the *Cxcr5* gene via homologous recombination. All mice were bred and maintained on a C57BL/6J mice background and maintained under specific-pathogen-free conditions. In some studies C57BL/Dk mice were also used, where indicated. All studies and regulatory licenses were approved by University of Edinburgh's ethics committee and carried out under the authority of a UK Home Office Project License.

### Genotype confirmation by PCR analysis.

CD11c^+^ cells were positively enriched from spleens by magnetic antibody cell sorting using CD11c microbeads according to the manufacturer's instructions (Miltenyi Biotec, Bisley, UK). The cells in the CD11c^−^ flowthrough fraction were further sorted using CD11b microbeads to enrich monocytes/macrophages, CD45R (B220) microbeads to enrich B cells, and CD90.2 microbeads to enrich T cells. DNA was extracted from each cell populations using a DNeasy blood and tissue kit (Qiagen, Crawley, UK) according to the manufacturer's instructions. RNA was extracted using RNA-Bee (AMS Biotechnology, Abingdon, UK), and cDNA was synthesized using a Superscript first-strand synthesis kit (Invitrogen). Where indicated, genomic or cDNA samples were analyzed for the presence of *Cre*, *Cxcr5*^F^, and recombined *Cxcr5*^F^ (*Cxcr5*^*de*-flox^) using the following primers: *Cre* (5′-CGAGTGATGAGGTTCGCAAGAACC-3′ and 5′-GCTAAGTGCCTTCTCTACACCTGC-3′) and *Cxcr5*^F^ and recombined *Cxcr5*^flox^ (*Cxcr5*^*de*-flox^; 5′-AGGAGGCCATTTCCTCAGTT-3′, 5′-GGCTTAGGGATTGCAGTCAG-3′, and 5′-TTCCTTAGAGCCTGGAAAAGG-3′).

### Macroscopic analysis of secondary lymphoid tissues.

Mice were injected intraperitoneally with 300 μl of 1% Chicago Sky Blue 6B in sterile phosphate-buffered saline. Mice were culled 7 days later, and the presence or absence of secondary lymphoid organs was determined macroscopically (41).

### Chemotaxis assays.

*Ex vivo* chemotaxis assays were performed as described previously (33, 95). Briefly, MLN cells in RPMI medium with 5% fetal calf serum (Invitrogen) were placed in the upper chamber of a 5-μm Transwell insert in a 24-well plate (Corning, Corning, NY). The lower chamber contained 200 ng of either CXCL13 or CCL21 (Peprotech, London, UK)/ml. Five technical replicates were performed. After 24 h, the upper chamber was discarded, and the cells in the lower chamber were collected and immunostained with anti-CD3 (clone 17A2) to detect T cells, anti-B220 (clone RA3-2GB) to detect B cells, and anti-CD11c (clone N418; BioLegend, London, UK) and resuspended in 500 μl of fluorescence-activated cell sorting buffer. The number of migrated cells was counted for 60 s using a LSRFortessa flow cytometer (BD Biosciences).

### Prion exposure and disease monitoring.

For oral exposure, mice were fed individual food pellets dosed with 50 μl of a 0.1% (wt/vol) dilution of scrapie brain homogenate (containing approximately 2.5 × 10^3^ i.c. 50% infectious dose [ID_50_] units) prepared from mice terminally affected with ME7 scrapie prions according to our standard protocol (4–6, 24, 29, 96). During the dosing period mice were individually housed in bedding- and food-free cages with water provided *ad libitum*. A single prion-dosed food pellet was then placed in the cage. The mice were returned to their original cages (with bedding and food *ad libitum*) as soon as the food pellet was observed to have been completely ingested. The use of bedding- and additional food-free cages ensured easy monitoring of consumption of the prion-contaminated food pellet. For i.c. exposure, mice were injected with 20 μl of a 1% dilution of scrapie brain homogenate. After prion exposure, mice were coded and assessed weekly for signs of clinical disease and culled at a standard clinical endpoint. The clinical endpoint of disease was determined by rating the severity of clinical signs of prion disease exhibited by the mice. The mice were clinically scored as “unaffected,” “possibly affected,” or “definitely affected” according to standard criteria that typically present in mice with terminal ME7 scrapie prion disease. Clinical signs following infection with the ME7 scrapie agent may include weight-loss, starry coat, hunched and jumpy behavior (at early onset) progressing to limited movement, upright tail, wet genitals, decreased awareness, discharge from eyes and/or blinking eyes, and ataxia of the hind legs. The clinical endpoint of disease was defined in one of the following ways: (i) the day on which a mouse received a second consecutive “definite” rating, (ii) the day on which a mouse received a third “definite” rating within four consecutive weeks, or (iii) the day on which a mouse was culled *in extremis*. Survival times were recorded for mice that did not develop clinical signs of disease during the observation period. Prion diagnosis was confirmed by histopathological assessment of vacuolation in the brain. For the construction of lesion profiles, vacuolar changes were scored in nine distinct gray-matter regions of the brain, as described previously (97).

For bioassay of prion infectivity, individual spleens were prepared as 1% (wt/vol) homogenates. For each tissue homogenate, groups of tga20 indicator mice (*n* = 4/homogenate) were injected i.c. with 20 μl of homogenate. The prion infectivity titer in each sample was determined from the mean incubation period in the indicator mice by reference to a dose/incubation period response curve for ME7 scrapie-infected spleen tissue serially titrated in tga20 mice using the following relationship: *y* = 9.4533 to 0.0595*x* (where *y* is the log ID_50_ U/20 μl of homogenate, and *x* is the incubation period [*R*^2^ = 0.9562]). Since the expression level of cellular PrP^C^ controls the prion disease incubation period, tga20 mice overexpressing PrP^C^ are extremely useful as indicator mice in scrapie agent infectivity bioassays because they succumb to disease with much shorter incubation times than conventional mouse strains (94).

### Splenectomy.

Groups of C57BL/Dk mice were surgically splenectomized and orally exposed to prions 8 days later. Briefly, an ∼1-cm incision was made in the left upper quadrant of the abdominal wall, and the spleen was identified. Blood supply to the organ was tied off with sutures before removal. The incision site was then also closed with sutures. Isoflurane anesthesia was used throughout the surgical procedure. After surgery, the mice were closely monitored throughout the recovery period and given buprenorphine hydrochloride as an analgesic. Sham-operated mice (laparotomy incision and suture repair with no spleen removal) were used as a control.

### IHC and immunofluorescent analyses.

Whole-mount immunostaining was performed as previously described (29). Briefly, Peyer's patches were fixed with BD Cytofix/Cytoperm (BD Biosciences, Oxford, UK), and subsequently immunostained with rat anti-mouse GP2 monoclonal antibody (MAb; MBL International, Woburn, MA). After the addition of primary antibody, the tissues were stained with Alexa Fluor 488-conjugated anti-rat IgG antibody (Invitrogen, Paisley, UK) and Alexa Fluor 647-conjugated phalloidin to detect f-actin (Invitrogen).

Intestines, MLN, and spleens were also removed and snap-frozen at the temperature of liquid nitrogen. Serial frozen sections (6 μm in thickness) were cut on a cryostat and immunostained with the following antibodies: FDC were visualized by staining with MAb 8C12 to detect CR1 (CD35; BD Biosciences) and cellular PrP^C^ was detected using PrP-specific polyclonal antibody (pAb) 1B3 (98). Where appropriate, sections were counterstained with DAPI (4′,6′-diamidino-2-phenylindole) to detect cell nuclei (Life Technologies).

For the detection of disease-specific PrP (PrP^d^) in intestines, MLN, spleens, and brains, tissues were fixed in periodate-lysine-paraformaldehyde fixative and embedded in paraffin wax. Sections (thickness, 6 μm) were deparaffinized and pretreated to enhance the detection of PrP^d^ by hydrated autoclaving (15 min, 121°C, hydration) and subsequent immersion in formic acid (98%) for 5 min. The sections were then immunostained with 1B3 PrP-specific pAb. For the detection of astrocytes, brain sections were immunostained with anti-glial fibrillary acidic protein (GFAP; Dako, Ely, UK). For the detection of microglia, deparaffinized brain sections were first pretreated with citrate buffer and subsequently immunostained with anti-ionized calcium-binding adaptor molecule 1 (Iba-1; Wako Chemicals GmbH, Neuss, Germany). For the detection of FDC in intestines, MLN, and spleens, deparaffinized sections were first pretreated with Target Retrieval Solution (Dako) and then immunostained with anti-CD21/35 (clone 7G6; BD Biosciences). PET immunoblot analysis was used to confirm the PrP^d^ detected by IHC was proteinase K-resistant PrP^Sc^ (49). Membranes were subsequently immunostained with 1B3 PrP-specific pAb.

For light microscopy, following the addition of primary antibodies, biotin-conjugated species-specific secondary antibodies (Stratech, Soham, UK) were applied, and immunolabeling was revealed using horseradish peroxidase-conjugated avidin-biotin complex (ABC kit; Vector Laboratories, Peterborough, United Kingdom) and visualized with DAB (diaminobenzidine; Sigma). Sections were counterstained with hematoxylin to distinguish cell nuclei. For fluorescence microscopy, following the addition of primary antibody, streptavidin-conjugated or species-specific secondary antibodies coupled to Alexa Fluor 488 (green), Alexa Fluor 594 (red), or Alexa Fluor 647 (blue) dyes (Life Technologies) were used. The sections were subsequently mounted in fluorescent mounting medium (Dako). Images of whole-mount immunostained intestinal pieces and cryosections were obtained using a Zeiss LSM710 confocal microscope (Zeiss, Welwyn Garden City, UK).

### Image analysis.

For morphometric analysis, images were analyzed using ImageJ software (http://rsb.info.nih.gov/ij/) as described on coded sections (42, 45, 99). Background intensity thresholds were first applied using an ImageJ macro which measures pixel intensity across all immunostained and nonstained areas of the images. The obtained pixel intensity threshold value was then applied in all subsequent analyses. Next, the number of pixels of each color (black, red, green, yellow, etc.) were automatically counted. For these analyses, data are presented as the proportion of positively stained pixels for a given IHC marker per total number of pixels (all colors) in the specific area of interest. In each instance, typically six images were analyzed per mouse, from tissues from multiple mice per group (*n* = 6 to 8 mice/group). Details for all of the sample sizes for each parameter analyzed are provided in the figure legends.

### Immunoblot detection of PrP^Sc^.

Brain homogenates (10% weight/volume) were prepared in NP-40 lysis buffer (1% NP-40, 0.5% sodium deoxycholate, 150 mM NaCl, 50 mM Tris-HCl [pH 7.5]) and incubated at 37°C for 1 h with proteinase K (PK) at 20 μg/ml. Digestions were halted by addition of 1 mM phenylmethylsulfonyl fluoride. Samples were then subjected to electrophoresis through 12% Tris-glycine polyacrylamide gels (Nupage; Life Technologies) and transferred to polyvinylidene difluoride membranes by semidry blotting. PrP was detected using anti-mouse PrP-specific MAb 7A12 (100), followed by horseradish peroxidase-conjugated goat anti-mouse antibody (Jackson ImmunoResearch) and visualized chemiluminescence (BM chemiluminescent substrate kit; Roche, Burgess Hill, UK).

### Statistical analyses.

Unless indicated otherwise, data are presented as means ± the standard errors (SE), and significant differences between samples in different groups were evaluated by Student *t* test. In instances where there was evidence of nonnormality, data were analyzed by nonparametric analysis of variance (Kruskal-Wallis test) with Dunn's multiple comparison post hoc test. *P* values of <0.05 were accepted as significant.
